# Effect of the Substrate Structure and Metal Ions on the Hydrolysis of Undamaged RNA by Human AP Endonuclease APE1

**DOI:** 10.32607/actanaturae.10864

**Published:** 2020

**Authors:** A. A. Kuznetsova, D. S. Novopashina, O. S. Fedorova, N. A. Kuznetsov

**Affiliations:** Institute of Chemical Biology and Fundamental Medicine, Siberian Branch, Russian Academy of Sciences, Novosibirsk, 630090 Russia

**Keywords:** human AP endonuclease, DNA repair, endoribonuclease activity, substrate specificity

## Abstract

Human apurinic/apyrimidinic (AP) endonuclease APE1 is one of the participants in the DNA base excision repair. The main biological function of APE1 is to hydrolyze the phosphodiester bond on the 5′-side of the AP sites. It has been shown recently that APE1 acts as an endoribonuclease and can cleave mRNA, thereby controlling the level of some transcripts. The sequences of CA, UA, and UG dinucleotides are the cleavage sites in RNA. In the present work, we performed a comparative analysis of the cleavage efficiency of model RNA substrates with short hairpin structures in which the loop size and the location of the pyrimidine–purine dinucleotide sequence were varied. The effect of various divalent metal ions and pH on the efficiency of the endoribonuclease reaction was analyzed. It was shown that site-specific hydrolysis of model RNA substrates depends on the spatial structure of the substrate. In addition, RNA cleavage occured in the absence of divalent metal ions, which proves that hydrolysis of DNA- and RNA substrates occurs via different catalytic mechanisms.

## INTRODUCTION

Human apurinic/apyrimidinic endonuclease APE1 is one of the most exhaustively studied DNA damage repair enzymes [1]. This enzyme cleaves the phosphodiester bond in DNA at the 5’-side from the AP site, resulting in rupturing of the ribose-phosphate backbone and formation of chain fragments carrying the 3’-hydroxy group and 2’-deoxyribose 5’-phosphate [2, 3]. However, the enzyme can recognize not only AP sites, but also some damaged nucleotides, such as 5,6-dihydrouridine, alpha anomer of adenosine, etc. [4]. Furthermore, APE1 exhibits 3’-phosphodiesterase, 3’-phosphatase [5], and 3’-5’-endonuclease activity [6, 7].

It was shown earlier that APE1 causes RNA chain degradation in DNA–RNA duplexes (i.e., exhibits RNase H activity) [8]. Later, it was discovered that APE1 can cleave both RNA containing the AP site [9] and undamaged highly structured mRNA (e.g., mRNA c-myc [10]). RNA-cleaving ability was also demonstrated for the miRNA, CD44 RNA, and RNA components of the SARS virus [11]. Undamaged RNA fragments are preferentially cleaved at the phosphodiester bond between the UA, UG, and CA dinucleotides in single-stranded sequences or weakly paired RNA regions, while the less hydrolyzable UC, CU, AC, and AU dinucleotides act as secondary cleavage sites [11]. Sequences rich in CA dinucleotides are known to be powerful splicing enhancers or silencers [12]; therefore, the preferential cleavage at CA sequences indicates that APE1 is possibly involved in mRNA splicing [13, 14].

Therefore, human AP endonuclease is versatile in terms of the functions it plays in the cell. Structural data, kinetic studies and a mutation analysis have made it possible to identify the key stages of interaction between APE1 and damaged DNA harboring the AP site [15, 16, 17], as well as some damaged [18] and undamaged [7] nucleotides. Kuznetsova et al. have suggested a mechanism for the broad substrate specificity of AP endonuclease exhibited upon its interaction with DNA [18]. For the catalysis to take place, special contacts form in the APE1–DNA complex. These contacts make the double helix of the damaged nucleotide unfold into the enzyme’s active site formed by the Asp308, His309, Glu96, Asp210, Tyr171, Asn212, and Asn174 residues. The amino acid residues of the enzyme preferentially interact with one strand of the double helix. In the catalytically competent enzyme–substrate complex, the phosphate group located on the 5’-side from the damaged nucleotide is coordinated by the Asn174, Asn212, and His309 residues. Phosphodiester bond hydrolysis starts with a nucleophilic attack on the phosphorus atom; the oxygen atom of a water molecule coordinated to the Asp210 residue through the Mg^2+^ ion acts as a nucleophile.

The role played by Mg^2+^ ions in the binding of damaged DNA, its cleavage, and release of the reaction product has been vigorously discussed in a number of studies [19-27]. Interestingly, the efficiency of phosphodiester bond hydrolysis in DNA containing an inactive AP site analog (2-oxymethyl-3-oxy-tetrahydrofuran, F site) decreases in the series Mg^2+^ > Mn^2+^ > Ni^2+^ > Zn^2+^ >> Ca^2+^, Cu^2+^ [16]. It seems that the Ca^2+^ ion, which is characterized by the largest ionic radius [28], cannot be properly coordinated to the metal-binding site of the enzyme and inhibits the catalytic process. Meanwhile, the inhibitory effect of Cu^2+^ ions is most likely related to the strong interaction between Cu^2+^ ions and nitrogenous bases, as well as phosphate groups as described earlier [29].

It is a known fact that APE1 does not need divalent metal ions to exhibit its endoribonuclease activity [11, 30]. Indeed, the RNA-cleaving properties of APE1 were observed both in 10 mM EDTA and in the presence of the Mg^2+^, Ca^2+^ and Mn^2+^ ions. Meanwhile, the endoribonuclease reaction was inhibited in the presence of the Zn^2+^, Ni^2+^, Cu^2+^ and Co^2+^ ions, as is the case for DNA substrates. Kim et al. [30] studied the activity of the mutant forms of APE1 carrying amino acid substitutions in the active site (N68A, D70A, Y171F, D210N, F266A, D283N, D308A and H309S) to elucidate the mechanism of endoribonuclease reaction. An analysis of the activities of these mutant APE1 forms with respect to the model RNA and DNA substrates demonstrated that most of the aforecited residues critical for AP site hydrolysis in double-stranded DNA also substantially contribute to endoribonuclease activity. The Asp283 residue is probably not involved in the hydrolysis of the RNA substrate, since the mutant APE1 form, D283N, retained its endoribonuclease activity in the absence of a metal ion. The key difference between the catalytic mechanisms of phosphodiester bond hydrolysis in RNA- and DNA substrates is that the reaction yileds different products: 3’-PO_4_^2-^ for RNA and 3’-OH for DNA [30].

Hence, according to the reported data, the catalytic mechanisms of DNA and RNA hydrolysis by the APE1 enzyme are different, but the reasons for that difference remain to be fully elucidated. Thus, it is unclear how the substrate spatial architecture and the nature of metal ions affect efficiency in endoribonuclease-catalyzed hydrolysis. Therefore, this study aimed to perform a kinetic analysis of endoribonuclease-catalyzed hydrolysis of the model RNA substrates that form hairpin structures carrying the CA or UA dinucleotide sequences at different loop or stem positions, with the loop length ranging from 2 to 5 nucleotides, in the absence or presence of divalent metal ions.

## EXPERIMENTAL

In this study, we used the following reagents, manufactured by Sigma-Aldrich (USA): acrylamide, N,N′-methylenebisacrylamide, dithiothreitol (DTT), urea, glycerol, HEPES, isopropyl-β-*D*-thiogalactopyranoside, Tris, NaCl, NaOH, EDTA, HCl, and divalent metal salts (CaCl_2_, CoCl_2_, MgCl_2_, MnCl_2_, ZnCl_2_, and NiSO_4_). All the solutions were prepared using twice-distilled water.

**APE1 enzyme**

The APE1 enzyme was isolated from *Escherichia coli* Rosetta 2 cells transformed with the plasmid pET11a harboring the human AP endonuclease gene, according to the procedure described earlier [15]. The *E. coli* Rosetta 2 cells were cultured in a LB medium (1 L) supplemented with 50 μg/ml ampicillin at 37°C until the optical density at 600 nm reached 0.6–0.7. The temperature was then reduced to 20°C, and transcription of the protein-coding insertion sequence was induced by addition of isopropyl-β-*D*-thiogalactopyranoside until a concentration of 0.2 mM was attained. After the induction, the cell culture was incubated for 16 h. The cells were precipitated by centrifugation for 10 min at 12 000 rpm, and a cell culture in 30 mL of the buffer solution (20 mM HEPES-NaOH, pH 7.8, 40 mM NaCl) was prepared. The cells were lysed with a French press. All the subsequent procedures were conducted at 4°C. The resulting cell lysate was centrifuged (40 min at 30 000 rpm); the supernatant was applied onto column I (Q-Sepharose Fast Flow, Amersham Biosciences, Sweden) and washed with a buffer solution consisting of 20 mM HEPES-NaOH, pH 7.8, and 40 mM NaCl. The fractions containing the APE1 protein were collected and applied onto column II (HiTrap-Heparin™, Amersham Biosciences). Chromatography was carried out in buffer solution I and a linear gradient of 40 → 600 mM NaCl; the optical density of the solution was recorded at 280 nm. The purity of the APE1 protein was determined by gel electrophoresis. The fractions containing the APE1 protein were subjected to dialysis in a buffer consisting of 20 mM HEPES-NaOH, pH 7.5, 1 mM EDTA, 1 mM DTT, 250 mM NaCl, and 50% glycerol and stored at -20°C. Enzyme concentration was calculated from the known optical density of the protein at 280 nm and the molar extinction coefficient (56,818 M-1×cm-1).

**Oligoribonucleotides**

Oligoribonucleotides were obtained via the solid-phase phosphite amide procedure on an ASM-800 synthesizer (Biosset, Russia) using the respective phosphite amides of 2’-O-*tert*-butyldimethylsilyl (2’-O-TBDMS) ribonucleotides (ChemGenes, USA). Fluorescein phophite amide (Glen Research, USA) was employed to insert a fluorescein tag at the 5’ end. Oligonucleotides carrying the BHQ1 fluorescence quencher at their 3’ end were obtained using a modified polymeric substrate, 3’-BHQ-1 CPG (Black Hole Quencher) (Glen Research, USA). Oligoribonucleotide deblocking was performed under standard conditions. The deblocked oligoribonucleotides were isolated by preparative PAGE (gel concentration, 15%) under denaturing conditions (acrylamide : N,N’-methylenebisacrylamide (30 : 1), 8 M urea, 50 mM Tris-H_3_BO_3_, pH 8.3, 0.1 mM EDTA). The gel bands containing the product were cut out, and the nucleotide material was eluted from the polyacrylamide gel. The crushed gel was placed into a 2.0- mL tube; 1–1.5 mL of 0.3 M LiClO_4_ was added; and the tubes were incubated at 25°C for 16 h under stirring in a thermomixer (Thermomixer Compact, Eppendorf, Germany). The oligonucleotides were desalinated on a C18 column (Waters, USA).

The homogeneity of the oligonucleotides and their derivatives was analyzed by PAGE (gel concentration, 15%) under the conditions described above. Prior to being applied on the gel, oligonucleotide samples (~ 0.05 arb. units) were supplemented with 4–5 μL of a 8 M urea solution containing 0.05% xylene cyanol FF and 0.05% bromophenol blue. A solution prepared using 50 mg of a Stains-all dye and 100 mL of a 1 : 1 formamide : a water mixture was used to visualize the oligonucleotides.

The optical density of the oligonucleotide solutions was measured on a NanoDrop 1000 spectrophotometer (ThermoScientific, USA) relative to deionized water. The molar extinction coefficient of the oligonucleotides or their conjugates at 260 nm was used to calculate oligonucleotide concentrations in the initial solution. The molar extinction coefficients of fluorescein-labeled oligoribonucleotide derivatives were taken equal to the sum of the molar extinction coefficients of the oligonucleotides and the molar extinction coefficient of fluorescein and the quencher tagged to the oligomer (20 900 M-1×cm-1 for FAM and 8 000 M-1×cm-1 for BHQ1). *Table 1* lists the sequences of the model oligoribonucleotides.

**Stopped-flow kinetic analysis**

The fluorescence kinetic curves were recorded on an SX.18MV stopped-flow spectrometer (Applied Photophysics, UK). The efficiency of the fluorescence resonance energy transfer (FRET) between the FAM/BHQ1 pair was measured by exciting the fluorescence of the FAM dye at 494 nm. The FRET signal was recorded at wavelengths above 530 nm using an OG 515 optical filter (Schott, Germany). The instrument dead time was 1.38 ms. Each kinetic curve was averaged over at least four experimental curves.

**Fluorescence titration**

Fluorescence titration was carried out on a Cary Eclipse fluorescence spectrophotometer. All the experiments were conducted during 10 min, so hydrolysis of the RNA substrates during titration was neglected. The enzyme solution was added to 100 μL of a 1.0 × 10-6 M solution of the RNA substrate in buffer solution (50 mM Tris-HCl (pH 7.5), 50 mM NaCl, 1 mM EDTA, 1 mM DTT, and 9% glycerol). The fluorescence emission spectrum of FAM was recorded at an excitation wavelength of 494 nm. For calculation of the dissociation constants, the experimental data were processed using the DynaFit software package (BioKin, Pullman, WA, USA) [31] employing the single-stage binding model.

**Microscale thermophoresis (MST)**

The stability constants of the complex formed between the substrates under study and the APE1 enzyme were determined on a Monolith NT.115 system (NanoTemper Technologies) using standard capillaries (MonolithTM NT.115 Standard Treated Capillaries). Each point on the titration curves was obtained by measuring the fluorescence intensities of individual solutions (10 μL) containing an oligonucleotide ligand (0.5 μM) and the enzyme (0.05–30 μM) in a buffer solution (50 mM Tris- HCl (pH 7.5), 50 mM KCl, 1 mM EDTA, 1 mM DTT, and 9% glycerol) at 25°C. For calculation of the dissociation constants, the experimental data were processed using the DynaFit software package (BioKin, Pullman, WA, USA) [31] employing the single-stage binding model.

**Kinetic analysis of the hydrolysis of RNA substrates**

**Table 1:** Model RNA substrates used in this study

RNA substrate, designation	Nucleotide sequence
HP1	5′ FAM-r(AUAUAAGAUUAUAU)-BHQ1 3′
HP2	5′ FAM-r(AUAUAAGAAUUAUAU)-BHQ1 3′
HP3	5′ FAM-r(AUAUAAGAUAUUAUAU)-BHQ1 3′
HP4	5′ FAM-r(AUAUAAGAUCAUUAUAU)-BHQ1 3′
HP5	5′ FAM-r(AUACAACAUAAUUGUAU)-BHQ1 3′
HP6	5′ FAM-r(AUAUAACAUCAUUAUAU)-BHQ1 3′
L	5′ FAM-r(AGAGAGGCAGAGA) 3′

Note.

^FAM^ – 6-carboxyfluorescein label; BHQ1 –black hole quencher.

^BHQ1^ – black hole quencher.

A kinetic analysis of the cleavage of our model RNA substrates was conducted using the following procedure. The enzyme (30 μL, 0.6–4 μM) in a buffer solution (50 mM Tris-HCl (pH 7.5), 50 mM KCl, 1 mM EDTA, 1 mM DTT, and 9% glycerol) was added to 30 μL of a buffer solution containing the substrate (2 μM) at 25°C. After the reaction mixture had been rapidly stirred, 10 μL aliquots were sampled in certain intervals. The reaction was stopped by adding 10 μL of the solution containing 9 M urea and 25 mM EDTA. PAGE (gel concentration, 20%) under denaturing conditions (7 M urea) was performed in a Protean II xi vertical thermostated electrophoresis chamber (Bio-Rad Laboratories, Inc., USA) at 55°C and a voltage of 200–00 V. The gel was visualized using an E-Box CX.5 TS gel documentation system (Vilber Lourman, France). The substrate cleavage efficiency was determined using the Gel-Pro Analyzer 4.0 (Media Cybernetics, USA). The cleavage efficiency was calculated as the ratio between the peak area of the cleavage product and the sum of peak areas of the product and the initial oligoribonucleotide. The putative error in determining the modification extent was usually ≤ 20%.

Partial hydrolysis of the RNase A substrate was performed using the following procedure. The reaction mixture (20 μL) containing the 3.0 μM substrate and 3.0 nM RNase A in a buffer solution (50 mM Tris-HCl (pH 8.5), 50 mM NaCl, 1 mM EDTA, 1 mM DTT, and 9% glycerol) was incubated at 25°C for 5 min. The reaction mixture was then supplemented with 20 μL of a solution containing 9 M urea and 25 mM EDTA and incubated at 96°C for 5 min.

## RESULTS AND DISCUSSION

**Design of RNA substrates**

**Fig. 1:**
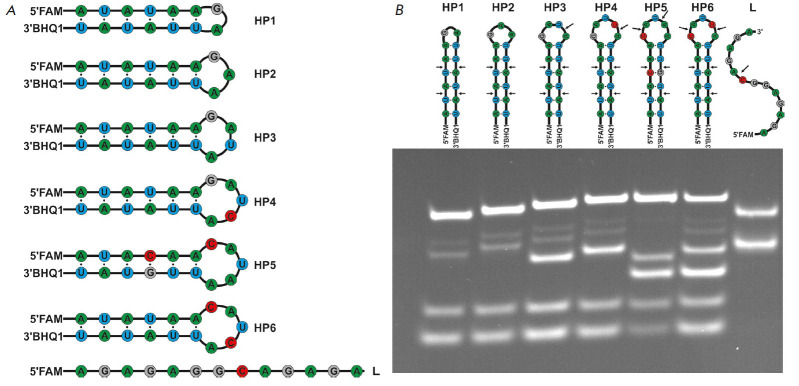
The structure of the RNA substrates used in this study (*A*). PAGE analysis of the cleavage of RNA substrates by RNase A. The positions of hydrolyzed nucleotides are indicated by arrows (*B*)

According to the reported data [10, 11], APE1 preferentially cleaves the CA, UA, and UG dinucleotide sequences in single-stranded or weakly paired RNA regions. Furthermore, weak RNA cleavage at the UC, CU, AC, and AU sites was also observed [11]. It is fair to assume that, along with the pyrimidine–purine sequence, the substrate structure (which can substantially affect recognition of the target site) is essential for the catalytic complex formation. Indeed, the RNA cleavage observed in single-stranded regions near the hairpin stems [10, 11] may be an indirect indication that a substantial contribution is made by the secondary structure of the substrate. Therefore, we used a series of 14- to 17-nucleotide-long model RNA substrates, the short hairpin structures in which the stem was 6 bp long and the loop length ranged from 2 to 5 nucleotides; the positions of the CA or UA dinucleotide in the hairpin loop or stem were also varied (*Fig. 1*). In order to study the kinetics of RNA substrate cleavage by FRET, the 5′- and 3′ ends of our model oligonucleotide were labeled with the FAM and BHQ1 dyes, respectively. The effect of the duplex portion of the hairpin on cleavage efficiency was evaluated using the 13-nucleotide-long linear substrate L carrying a single CA dinucleotide. Hydrolysis specificity with respect to pyrimidine–purine dinucleotides was controlled using RNase A, which is specific to cleaving pyrimidine nucleotides regardless of their structural position (*Fig. 1*).

*Figure 1* shows that bands corresponding to the products of the cleavage at four UA sequences in the stem are observed for all the substrates. Furthermore, additional bands corresponding to the cleavages at UA and CA in the loops appear for the HP3–HP6 substrates.

**The effect of divalent metal ions and pH on the interaction between the APE1 and RNA substrates**

**Fig. 2:**
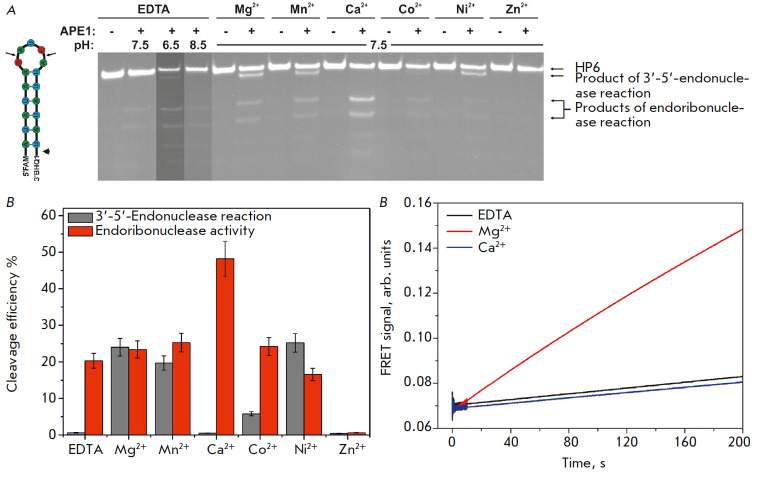
Cleavage of the HP6 substrate by APE1 in the presence of different divalent metal ions and at different pH. (*A*) PAGE analysis of the reaction products. The positions of hydrolyzed nucleotides are indicated by arrows. (*B*) Comparison of the cleavage efficiencies of HP6 by APE1 in the presence of different divalent metal ions. [APE1] = 2 μM, [RNA] = 1 μM, [EDTA/Me^2+^] = 1/5 mM, T = 25°C, reaction time = 1 h. (*C*) Stopped-flow FRET-signal traces for HP6, [APE1] = 2 μM, [RNA] = 1 μM, [EDTA/Me^2+^] = 1/5 mM; T = 25°C

It was established for the HP6 RNA substrate that 3′-5′-exonuclease and endoribonuclease reactions occur in the presence of the Mg^2+^, Mn^2+^ and Ni^2+^ ions, whereas products of the endoribonuclease reaction preferentially accumulate in the presence of the Ca^2+^ and Co^2+^ ions (*Figs. 2A and B*). Meanwhile, the enzyme exhibited no activity in the presence of Zn^2+^ ions. In addition, the yield of hydrolysis products dropped noticeably when pH of the EDTA-containing buffer was increased to 8.5, while pH reduction to 6.5 produced side products due to statistical hydrolysis.

We compared the kinetic curves (*Fig. 2C*) characterizing the interaction between APE1 and HP6 and came to the conclusion that the initial rates of the endoribo nuclease reaction in the presence of EDTA or Ca^2+^ ions are similar, while the fluorescence intensity of FAM in the presence of Mg^2+^ ions increases more rapidly (presumably due to the appearance of the 3’-5’ endonuclease reaction). It is noteworthy that the changes in the FAM fluorescence intensity in the presence of Mg^2+^ ions can also be related to the formation of a catalytic exonuclease complex, which increases the distance between the BHQ1 quencher (residing at the 3’ end) and the 5’-end FAM label.

Interestingly, when APE1 interacts with the DNA substrate carrying the F site, its activity increases with pH, while the catalytic reaction is completely inhibited in the absence of divalent metal ions or in the presence of Ca^2+^ ions [16]. The presence of products of endoribonuclease activity under similar conditions demonstrates that hydrolysis of RNA substrates proceeds via an alternative metal-independent catalytic pathway.

According to these findings (*Fig. 2*), three buffer solutions (pH 7.5) containing 5 mM MgCl_2_, 5 mM CaCl_2_, or 1 mM EDTA were used to analyze various types of enzyme activity in further experiments with different RNA substrates.

**Hydrolysis of RNA substrates by APE1 in the presence of EDTA**

**Fig. 3:**
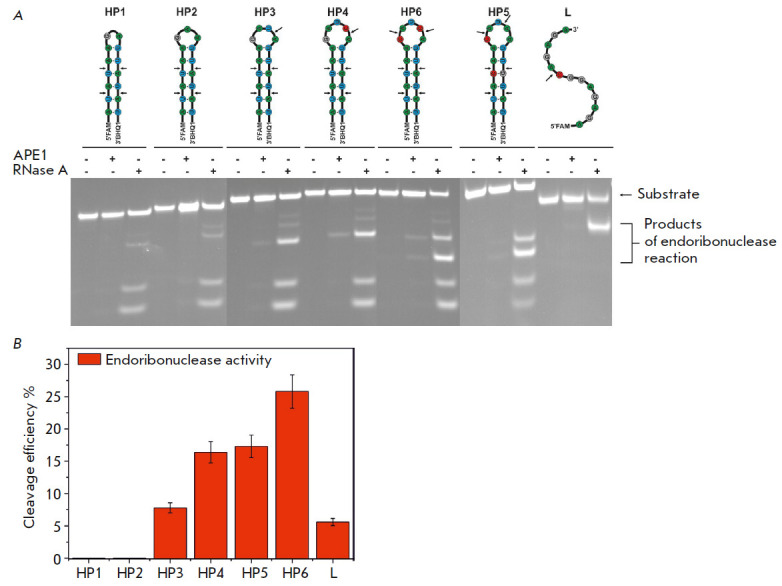
Cleavage of RNA substrates by APE1 in the presence of 1 mM EDTA. (*A*) PAGE analysis of the reaction products. The positions of hydrolyzed nucleotides are indicated by arrows. (*B*) Comparison of the cleavage efficacy of RNA substrates by APE1; [APE1] = 2 μM, [RNA] = 1 μM, T = 25°C, reaction time = 1 h

The interaction between APE1 and our model RNA substrates in the absence of divalent metal ions causes the accumulation of products of the endoribonuclease reaction for HP3–HP6 RNA substrates carrying the CA or UA dinucleotide in the loop portion (*Fig. 3*). Indeed, comparison of the sites of cleavage of the RNA substrates of RNase A, which statistically cleaves RNA at all pyrimidine nucleotides, indicates that the UA dinucleotides within the duplex portion of a hairpin are not recognized by AP endonuclease as cleavage sites. Meanwhile, in the absence of divalent metal ions, APE1 does not catalyze the 3′-5′-exonuclease reaction with any of the RNA substrate under study (*Fig. 3*).

Interestingly, the HP6 hairpin harboring two CA cleavage sites becomes the most efficient substrate, its cleavage efficiency being as high as 25% (*Fig. 3B*). Meanwhile, PAGE has demonstrated that both sites are characterized by similar cleavage efficiencies, thus proving that this parameter is independent of the position occupied by the CA dinucleotide within the loop. Meanwhile, when APE1 interacts with HP4 carrying a single CA dinucleotide, cleavage efficiency reaches 16%. The HP5 hairpin carries the UA and CA dinucleotides in its loop; the total cleavage efficiency is 17%. However, cleavage at the UA dinucleotide is much less efficient than cleavage at the CA dinucleotide (*Fig. 3*), which is also consistent with the low (8%) cleavage efficiency of HP3 carrying a single UA dinucleotide. However, when comparing the cleavage efficiencies of these substrates, one needs to take into account the substrate structure (different loop sizes and different positions of the hydrolyzed bond within the loop) in addition to the CA/UA context of the hydrolyzed phosphodiester bond. Since the cleavage site in the HP3 (UA) and HP4 (CA) hairpins has the same location with respect to the stem, it is fair to assume that the HP4 hairpin loop containing five nucleotides is more readily adapted in the enzyme substrate-binding site compared to the HP3 hairpin with the 4-nucleotide loop. The HP5 hairpin, whose loop is also five nucleotides long, differs from HP4 in the positions of cleavage sites, which presumably impedes efficient formation of the catalytic complex for HP5.

The linear substrate is also characterized by low cleavage efficiency (~ 5%), which indicates that the double-stranded portion of RNA substrates plays a crucial role in the formation of the catalytic enzyme– substrate complex.

**Table 2:** The values of the dissociation constant K_d_

Substrate	Buffer	K_d_, μM	
		Fluorescence titration	MST
HP1	EDTA	13.5 ± 8.2	12.1 ± 4.3
HP2	–«–	7.8 ± 5.5	4.6 ± 0.9
HP3	–«–	4.9 ± 1.1	2.6 ± 0.6
HP4	–«–	3.0 ± 0.7	3.6 ± 1.3
HP5	–«–	3.1 ± 1.0	2.5 ± 0.6
HP6	–«–	2.2 ± 0.5	2.7 ± 0.5
	Mg2+	2.8 ± 0.6	–
	Ca2+	1.6 ± 0.3	–
	Mn2+	3.6 ± 0.8	–
L	EDTA	1.5 ± 0.3	–

**Binding of RNA substrates to APE1 in the presence of EDTA**

**Fig. 4:**
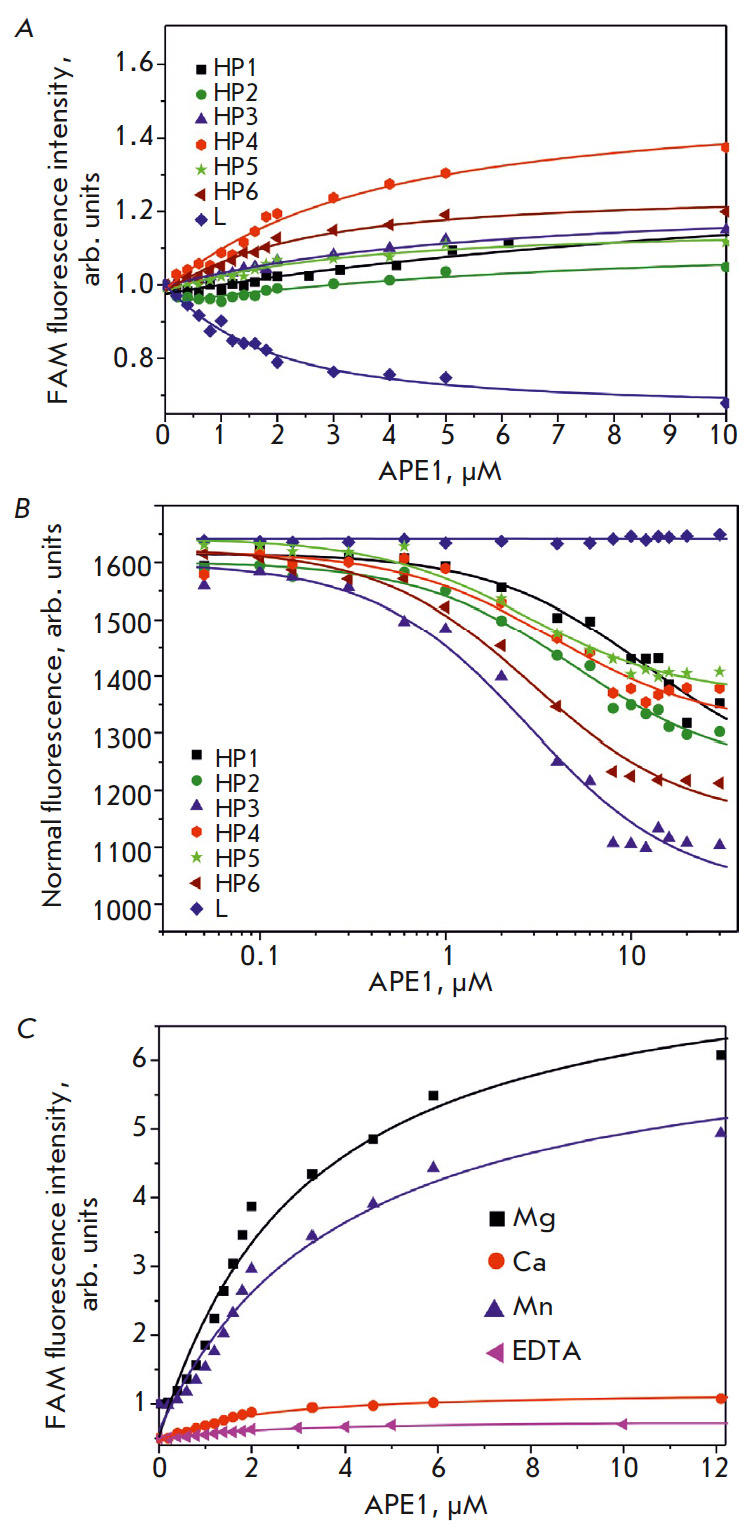
Determining the dissociation constant of enzyme– substrate complexes by fluorescence titration (*A, C*) and microscale thermophoresis (*B*)

The low cleavage efficiency of RNA substrates during a 1-h reaction (*Fig. 3*) allowed us to carry out experiments involving fluorescent titration of the substrates by the enzyme and estimate the dissociation constant of the enzyme–substrate complex. *Figure 4A* shows the fluorescence intensity at 520 nm as a function of enzyme concentration. For the HP1–HP6 substrates labeled with the FAM/BHQ1 pair, the FRET signal increased as the enzyme–substrate complex formed. Fluorescence quenching was observed in the case of linear substrate L harboring only the 5’-FAM label. Such differing changes in the FAM fluorescence intensity can be attributed to the fact that the enzyme can bind both to the loop and the 5’/3’-end of the hairpin structures. The presence of products of endoribonuclease hydrolysis in the reaction mixtures indicates that binding occurred in the loop portion of the substrate, while the presence of the products of 3′-5′-exonuclease degradation proves that an alternative complex with the 5’/3’-end region formed. Since the BHQ1 quencher resides at the 3’ end of the hairpins, the higher fluorescence intensity of FAM for the HP1–P6 substrates harboring the FAM/BHQ1 pair can be attributed to the fact that the distance between the FAM and BHQ1 labels increases when the enzyme–ubstrate complex with the 5’/3’-end region is formed. In the case of substrate L, which does not harbor the BHQ1 quencher, the fluorescence intensity of FAM decreases as a complex with an enzyme molecule is formed. The values of the dissociation constant *K*d were calculated (*Table 2*).

An additional microscale thermophoresis (MST) study of the binding between APE1 and the substrates was carried out (*Fig. 4B*), and the respective dissociation constants *K*d were calculated (*Table 2*). However, the titration curve for substrate L recorded by MST did not allow us to determine the *K*d value using this method because of the low signal-to-noise ratio. A comparison of the dissociation constants (*Table 2*) revealed rather good agreement between the values obtained using different methods. Hence, an analysis of the stability of the enzyme–substrate complexes using fluorescence titration and MST demonstrates that the highest dissociation constants were observed for the complex that formed between APE1 and HP1 or HP2, which contain a short loop and carry no specific dinucleotide in their loop. The complexes formed between the enzyme and the HP3–HP6 substrates have similar dissociation constants lying within the range of 2.2–4.9 μM.

We also estimated the dissociation constants of the complex containing HP6 as a substrate in the presence of divalent metal ions (*Fig. 4C*, *Table 2*). Strong quenching of substrate fluorescence in the presence of Co^2+^, Ni^2+^, and Zn^2+^ made it impossible to determine the *K*d value, whereas reduction of the *K*d value in the presence of Ca^2+^ may have been an indication that the enzyme–substrate complex had been stabilized.

**Hydrolysis of RNA substrates by APE1 in the presence of Ca^2+^ ions**

**Fig. 5:**
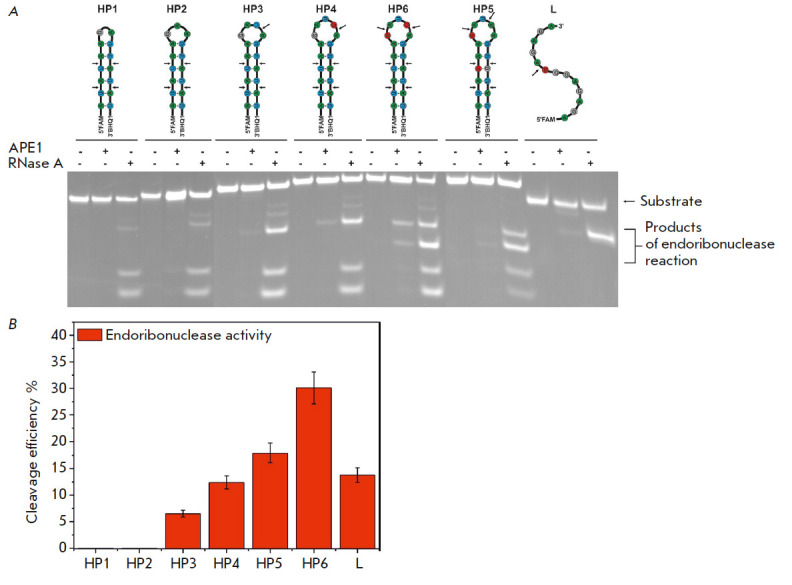
Cleavage of RNA substrates by APE1 in the presence of 5 mM CaCl_2_. (*A*) PAGE analysis of the reaction products. The positions of hydrolyzed nucleotides are indicated by arrows. (*B*) Comparison of the cleavage efficiencies of RNA substrates by APE1; [APE1] = 2 μM, [RNA] = 1 μM, T = 25°C, reaction time = 1 h

The interaction between APE1 and RNA substrates in the presence of Ca^2+^ ions increased efficiency in hydrolysis at specific CA sites within the loop portion of the HP6 hairpin and in the linear substrate, compared to that in the absence of divalent metal ions (*Fig. 5*). It is interesting to note that in the case of HP4, Ca^2+^ ions slightly reduced the APE1 activity, while exhibiting no effect when HP3 and HP5 were used as substrates. These effects are presumably caused by the fact that Ca^2+^ ions have a small impact on the dissociation constant of the enzyme–substrate complex, which agrees with the fluorescence titration data (*Table 2*).

**Fig. 6:**
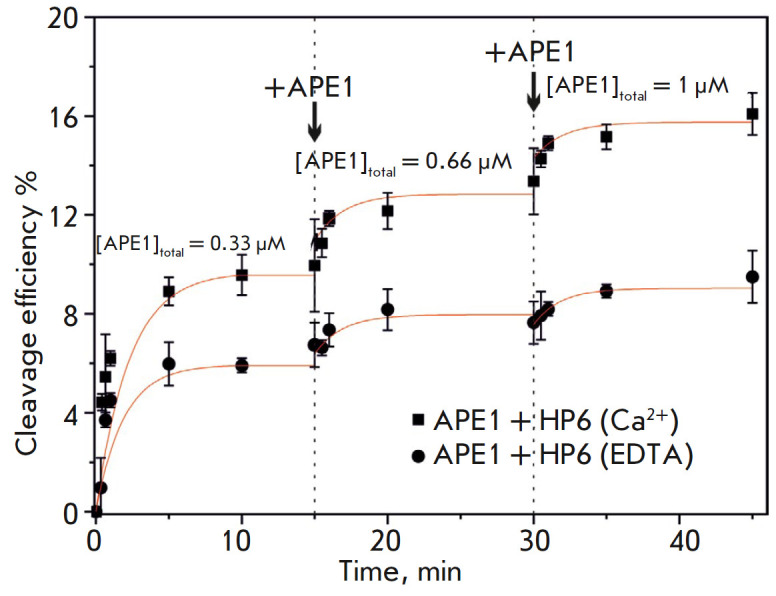
The time-course of product accumulation during cleavage of HP6 in the presence of 1 mM EDTA or 5 mM CaCl_2_ as revealed by PAGE, [RNA] = 1 μM

The kinetics of accumulation of the products of APE1 endoribonuclease activity on the HP6 RNA substrate in the absence of divalent metal ions and in the presence of Ca^2+^ ions were recorded (*Fig. 6*). Interestingly, when a threefold excess of the substrate was chosen, its cleavage efficiency reached a plateau within 15 min (~ 5 and 9% in the presence of EDTA and CaCl_2_, respectively). The low cleavage efficiency may indicate that: (1) efficiency in the catalytic complex formation is low and (2) the enzyme becomes strongly bound to the reaction product. Indeed, the new portion of the enzyme added to the reaction mixture led to additional accumulation of the reaction products, which demonstrates that APE1 remains bound to the RNA product after a single catalytic event.

**Hydrolysis of RNA substrates by APE1 in the presence of MgCl_2_**

**Fig. 7:**
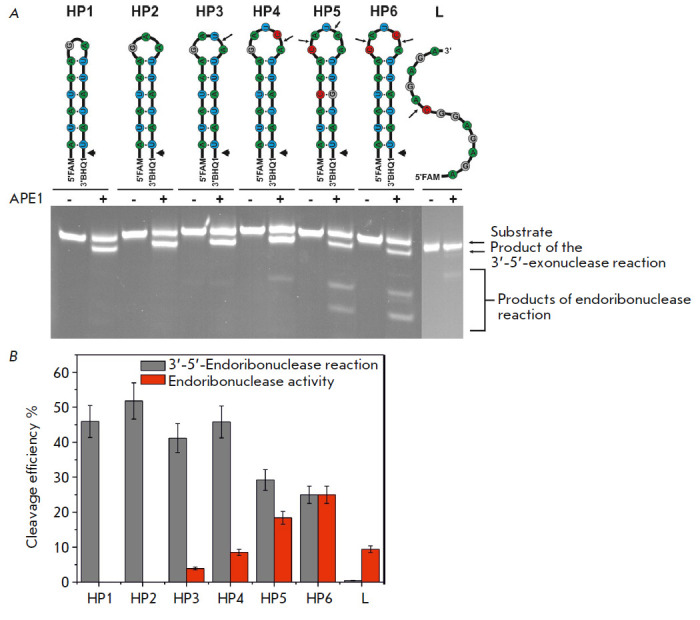
Cleavage of RNA substrates by APE1 in the presence of 5 mM MgCl_2_. (*A*) PAGE analysis of the reaction products. The positions of hydrolyzed nucleotides are indicated by arrows. (*B*) Comparison of cleavage efficiencies of RNA substrates by APE1, [APE1] = 2 μM, [RNA] = 1 μM, T = 25°C, reaction time = 1 h

Products of both the endoribonuclease and 3’-5’-exoribonuclease reactions accumulated when APE1 interacted with RNA substrates in the buffer containing MgCl_2_ (*Fig. 7*). The endoribonuclease reaction products did not form when HP1 and HP2 substrates with the shortest loop (2 and 3 nucleotides, respectively) that did not carry the specific pyrimidine–purine sequence were used. The products formed on the HP3–HP6 substrates due to the endoribonuclease activity corresponded to cleavage of the CA and UA dinucleotides within the loop. The 3′-5′-exoribonuclease reaction was found to occur on all the hairpin RNA structures harboring 3′-BHQ1. In this reaction, 3′-BHQ1 was removed and a typical increase in the mobility of the exoproduct in PAGE was observed (*Fig. 7*).

**Fig. 8:**
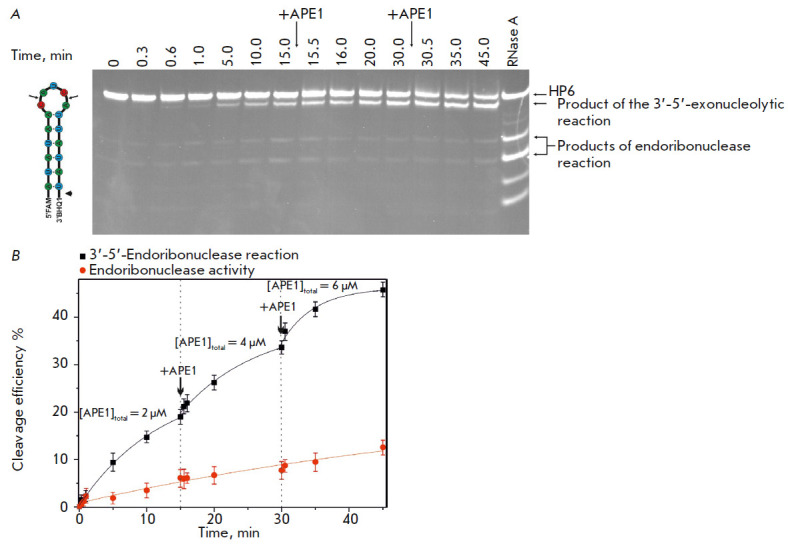
The time-course of product accumulation during cleavage of HP6 in the presence of 5 mM MgCl_2_. (*A*) PAGE analysis of the reaction products, [RNA] = 1 μM. (*B*) The time-course of accumulation of the cleaved product.

An analysis of the accumulation kinetics of the products of the endo- and exonuclease reactions of RNA-substrate conversion in the presence of Mg^2+^ ions demonstrated that cleavage of the 3′-BHQ1 is more efficient than cleavage within the loop (*Fig. 8*). Meanwhile, when the enzyme was returned to the reaction mixture, the rate of product accumulation increased for the exonuclease reaction but not for the endonuclease one (*Fig. 8B*). This difference may be an indication that the hairpin harbors several enzymebinding sites. AP endonuclease is tightly bound to the loop portion of the hairpin in order to slowly perform the endonuclease reaction. In this case, the additional enzyme introduced into the reaction mixture results in additional binding only to the vacant opposite end carrying the FAM/BHQ1 dyes, which is accompanied by the exonuclease reaction. It can also be assumed that the catalytic complex that forms between APE1 and the 5’/3’ end of HP6 in the presence of Mg^2+^ ions causes steric hindrance in the formation of the catalytic complex with the loop.

## CONCLUSION

In this study, we have analyzed the interaction between human AP endonuclease APE1 and model RNA substrates with different structures. APE1 was shown to efficiently bind both to the linear RNA substrate and to the RNA substrates forming the hairpin. The endoribonucleolytic cleavage of the substrates took place in the loop fragments at the CA and UA sequences. The efficiency of cleavage at the UA dinucleotide is lower than at the CA dinucleotide. However, when comparing the cleavage efficiencies of these substrates, one needs to take into account not only the CA/UA nucleotide context of the hydrolyzed phosphodiester bond, but also the substrate structure (including both the loop size and the position of the bond being hydrolyzed within the loop). Since in the HP3 (UA) and HP4 (CA) hairpins the cleavage site occupies the same position with respect to the hairpin stem, these findings allow one to assume that the 5-nucleotide-long HP4 hairpin loop is more readily adapted in the substrate-binding site of the enzyme compared to the HP3 hairpin with the 4-nucleotide-long loop. The HP5 (CA/UA) hairpin, whose loop also consists of five nucleotides, differs from HP4 in terms of cleavage site positions, which presumably impedes efficient catalytic complex formation in the case of HP5. Hence, a conclusion can be drawn that the formation of a catalytic enzyme–substrate complex depends both on the conformational strain of the loop in the hairpin-shaped RNA substrate and on the context and position of the phosphodiester bond to be hydrolyzed. It is worth mentioning that cleavage efficiency of linear substrate L was also low compared to that of the HP4–HP6 hairpin substrates. Given this fact, it is fair to assume that the structured duplex portion of the hairpin plays a crucial role in the formation of nonspecific contacts in the enzyme’s active sites, which are essential for catalytic complex formation.
